# Advances in hepatocellular carcinoma drug resistance models

**DOI:** 10.3389/fmed.2024.1437226

**Published:** 2024-07-31

**Authors:** Yien Xiang, Jun Wu, Hanjiao Qin

**Affiliations:** ^1^Department of Hepatobiliary and Pancreatic Surgery, the Second Hospital of Jilin University, Changchun, China; ^2^Department of Radiotherapy, the Second Hospital of Jilin University, Changchun, China

**Keywords:** hepatocellular cell carcinoma, *in vitro* model, *in vivo* model, drug resistance, patient-derived xenograft

## Abstract

Hepatocellular carcinoma (HCC) is the most common primary liver cancer. Surgery has been the major treatment method for HCC owing to HCC’s poor sensitivity to radiotherapy and chemotherapy. However, its effectiveness is limited by postoperative tumour recurrence and metastasis. Systemic therapy is applied to eliminate postoperative residual tumour cells and improve the survival of patients with advanced HCC. Recently, the emergence of various novel targeted and immunotherapeutic drugs has significantly improved the prognosis of advanced HCC. However, targeted and immunological therapies may not always produce complete and long-lasting anti-tumour responses because of tumour heterogeneity and drug resistance. Traditional and patient-derived cell lines or animal models are used to investigate the drug resistance mechanisms of HCC and identify drugs that could reverse the resistance. This study comprehensively reviewed the established methods and applications of *in-vivo* and *in-vitro* HCC drug resistance models to further understand the resistance mechanisms in HCC treatment and provide a model basis for possible individualised therapy.

## 1 Introduction

Primary liver cancer is a common digestive system malignancy with extremely high rates of incidence and mortality, ranking sixth and fourth, respectively (1). Primary liver cancer includes hepatocellular carcinoma (HCC), intrahepatic cholangiocarcinoma and combined hepatocellular cholangiocarcinoma, with HCC as the most common, accounting for almost 90% of cases (1). Surgery remained the most important treatment method for HCC over the years owing to HCC’s poor sensitivity to chemotherapy. However, postoperative patients show a high risk of recurrence or metastasis. Moreover, many HCC patients are diagnosed with advanced tumours and have lost the opportunity for surgery. In recent years, with the gradual popularisation of new treatment techniques, such as radiofrequency, microwave, freezing and TACE, and the development of numerous targeted and immunotherapeutic drugs, the progression-free and overall survival rates of HCC have greatly improved. However, primary and acquired drug resistance to these medications remains the most critical challenge in HCC treatment. This study reviewed original articles about drug resistance of HCC published in the last 5 years. The drug resistance models that were employed are presented, as well as a detailed introduction to some of the major drug resistance mechanisms that were discovered utilising the drug resistance models.

## 2 Traditional *in vitro* and *in vivo* drug resistance models

### 2.1 Establishment methods of traditional drug resistance models

**Figure 1:**
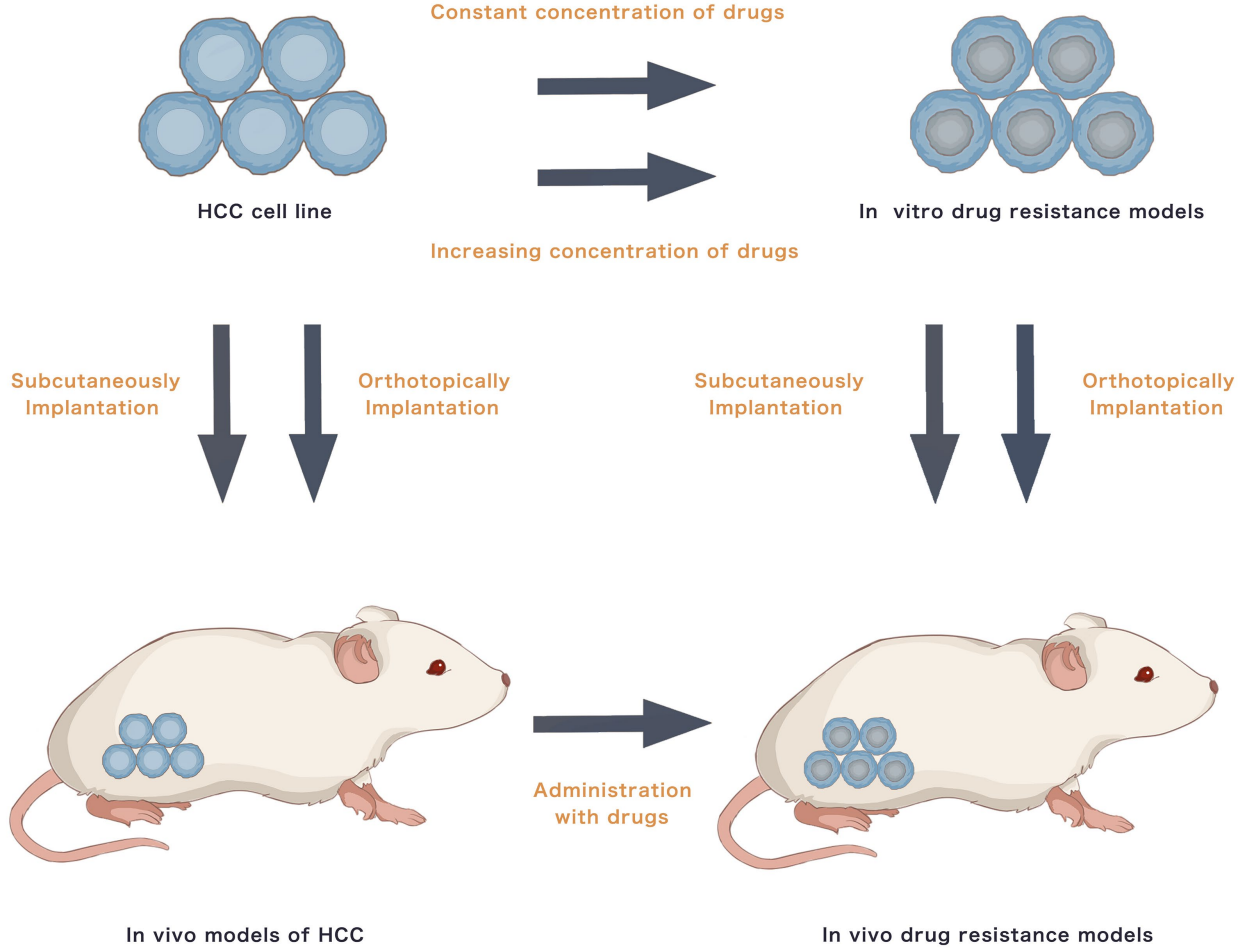
Hepatocellular carcinoma traditional drug resistance models.

Most studies have used traditional commercial HCC cell lines to establish drug resistance models. *In-vitro* models involve exposing parental HCC cells to medications at either a continuous high or a progressively increasing concentration for 3–6 months or 20–30 generations for the cells to develop drug-specific resistance. For *in-vivo* drug resistance models, drug-resistant RCC cells can directly be implanted subcutaneously or orthotopically into nude mice. Furthermore, parental RCC cells may be injected subcutaneously or orthotopically into nude mice, followed by prolonged oral drug feeding to acquire drug resistance. Figure 1 presents the establishment methods of traditional drug resistance models. Table 1 presents studies on HCC drug resistance using traditional *in-vitro* and *in-vivo* models. Briefly, HepG2, Huh7, SMMC-7721, MHCC97H, MHCC97L, Hep3B, BEL-7402, PLC/PRF/5 and SK-Hep-1 are commonly used to induce acquired drug resistance. Traditional chemotherapy drugs, such as cisplatin, oxaliplatin, 5-FU and doxorubicin, and TKIs, including sorafenib, lenvatinib and regorafenib, are major research topics. Numerous signalling pathways may be involved in resistance to a single agent. A specific drug that could reverse the drug resistance-associated signalling may present its potential as an alternative or combined treatment.

**Table 1:** Researches on HCC drug resistance using traditional *in-vitro* and *in-vivo* models.

Year	Model	Primary/ secondary	Resisted drug	*In vitro*/ vivo	Core molecule	Combined drug	Reference
2019	HepG2	Secondary	Cisplatin	*In vitro*	PKA/ PP2 A/ IKK pathway	Andrographolide	(2)
2019	HepG2, Huh7	Secondary	Sorafenib	*In vitro*, vivo	LncRNA SNHG1	–	(3)
2019	SMMC-7721	Secondary	Sorafenib	*In vivo*	M2 macrophage	–	(4)
2019	HepG2, HUH7	Secondary	Sorafenib	*In vitro*	miR-150-5p	lncRNA FOXD2-AS1	(5)
2019	HepG2, Huh7, SMMC-7721	Secondary	Oxaliplatin	*In vitro*	Connexin 32	–	(6)
2019	MHCC97H, Hep3B	Secondary	Oxaliplatin	*In vitro*, vivo	CCN2/ MAPK/ Id-1	–	(7)
2019	HepG2	Secondary	Sorafenib	*In vitro*	Rb, p16	Ribociclib	(8)
2019	HA22T	Secondary	Apicidin, SAHA	*In vitro*, vivo	PP1, eIF2α	Fisetin	(9)
2019	BEL-7402	Secondary	5-FU	*In vitro*	–	AC10364	(10)
2019	HepG2	Secondary	Cisplatin	*In vitro*	PARP1, HMGB1	Morin hydrate	(11)
2019	BEL-7402	Secondary	5-FU	*In vitro*, vivo	YAP	–	(12)
2019	HuH7	Secondary	Sorafenib	*In vitro*, vivo	miR-16, 14–3-3η	–	(13)
2019	HepG2, PLC/PRF/5, JHH-6	Secondary	Everolimus	*In vitro*	MiR-375	Vitamin D	(14)
2019	BEL-7402	Secondary	5-FU	*In vitro*	PLCβ3	–	(15)
2019	Hep3B, Huh7	Secondary	Sorafenib	*In vitro*	EGFR, KLF4	–	(16)
2019	BEL-7402	Secondary	5-FU	*In vitro*	MDR1, ABCC1, ABCG2	Parthenolide-5-FU conjugate	(17)
2019	HepG2	Secondary	Sorafenib	*In vitro*	Vimentin	–	(18)
2019	Ba/F3, Hep3B	Secondary	Fisogatinib	*In vitro*, vivo	On-target FGFR4 kinase domain mutations	Gatekeeper-agnostic, pan-FGFR inhibitor	(19)
2019	Hep3B	Secondary	Paclitaxel	*In vitro*	P-gp	Achillin	(20)
2019	HepG2, Huh7	Secondary	Sorafenib	*In vitro*, vivo	STAT-3	Phloretin	(21)
2019	HepG2	Secondary	Sorafenib	*In vitro*, vivo	Hypoxic microenvironment	Plantamajoside	(22)
2019	Hep3B, HuH7	Secondary	Sorafenib	*In vitro*, vivo	α-fetoprotein, glypican 3, survivin	Compound 9a	(23)
2019	Hep3B, HepG2	Secondary	Sorafenib	*In vitro*	DNMT3b, OCT4	–	(24)
2019	MHCC97H	Secondary	Oxaliplatin	*In vitro*, vivo	HSCs remodeling	–	(25)
2019	MHCC97H, Hep3B	Secondary	Oxaliplatin	*In vitro*, vivo	HSF1, AMPKα2	–	(26)
2020	MHCC97L, Hep3B	Secondary	Oxaliplatin, saracatinib	*In vitro*	Wnt-ABCG1 signalling	–	(27)
2020	HepG2	Secondary	Sorafenib	*In vitro*, vivo	Apoptosis	BEZ235	(28)
2020	Huh7, SMMC-7721	Secondary	Sorafenib	*In vitro*, vivo	EZH2	–	(29)
2020	HuH7, Hep3B	Secondary	Sorafenib	*In vitro*	LncRNA DANCR	–	(30)
2020	Hep3B	Secondary	Paclitaxel	*In vitro*	FOXO6	–	(31)
2020	Bel7402	Secondary	5-FU	*In vitro*	Amyloid precursor protein	–	(32)
2020	Huh1, Huh7	Secondary	Sorafenib	*In vitro*, vivo	Capicua	–	(33)
2020	Mahlavu, Hep3B, Huh7	Secondary	Radiotherapy	*In vitro*	PDK1	–	(34)
2020	Bel7402	Secondary	5-FU	*In vitro*	89 methylation forms	–	(35)
2020	HuH-7	Secondary	Sorafenib	*In vitro*, vivo	EphA2	–	(36)
2020	HepG2, HuH-7	Secondary	Sorafenib	*In vitro*	HANR	–	(37)
2020	HepG2, SMMC-7721	Secondary	Sorafenib	*In vitro*, vivo	LncRNA MALAT1	–	(38)
2020	HepG2	Secondary	Oxaliplatin	*In vitro*	CircFBXO11	–	(39)
2020	HepG2, HuH-7	Secondary	Sorafenib	*In vitro*	circRNAs	–	(40)
2020	HuH-7	Secondary	Sorafenib	*In vitro*	LRP8	–	(41)
2020	SK-Hep-1, HepG2	Secondary	Sorafenib	*In vitro*, vivo	miR-486-3p	–	(42)
2020	Hep3B	Secondary	Sorafenib	*In vitro*, vivo	SNGH16	–	(43)
2020	Bel7402	Secondary	5-FU	*In vitro*	Rab27B	–	(44)
2020	HuH7, HepG2	Secondary	Doxorubicin, Sorafenib	*In vitro*	AMPK	TFAM	(45)
2020	HuH7, Hep3B	Secondary	Sorafenib	*In vitro*	–	BA-5	(46)
2020	SK-HEP-1, HepG2	Secondary	Adriamycin	*In vitro*, vivo	CircFoxo3	–	(47)
2020	HepG2, MCF-7	Secondary	Adriamycin	*In vitro*	JNK2	GL-V9	(48)
2020	HUH7, RIL175	Secondary	Sorafenib	*In vitro*, vivo	Mitochondrial dysfunction	Tigecycline	(49)
2020	SMCC-7721, MHCC97	Secondary	Regorafenib	*In vitro*, vivo	SphK2	–	(50)
2020	SK-Hep1, Huh7	Secondary	Sorafenib	*In vitro*, vivo	Jagged2, Notch1	Valproic acid	(51)
2020	HepG2	Secondary	Doxorubicin	*In vitro*	HCSP4-Lipo-DOX-miR101	–	(52)
2020	HCCLM3, Huh7	Secondary	Sorafenib	*In vitro*, vivo	LncRNA HEIH	–	(53)
2020	HepG2, Huh7	Secondary	TRAIL	*In vitro*, vivo	C-Met, cyclin B1	–	(54)
2020	SMMC7721	Secondary	Sorafenib	*In vitro*	HDAC11	–	(55)
2020	Huh7	Secondary	Sorafenib	*In vitro*, vivo	JAK/STAT, PI3K/AKT, ERK/MAPK	Fostamatinib	(56)
2020	SK-HEP-1, Huh-7	Secondary	Sorafenib	*In vitro*	KCNQ1OT1	–	(57)
2020	Huh7, Hep3B	Secondary	Sorafenib	*In vitro*	mTORC2-AKT-BAD pathway	Torin2	(58)
2020	HepG2, Huh7	Secondary	Sorafenib	*In vitro*, vivo	miR-30a-5p, CLCF1	–	(59)
2020	HepG2, Huh7	Secondary	Sorafenib	*In vitro*	PD-L1	–	(60)
2020	HepG2, Huh7	Secondary	Sorafenib	*In vitro*, vivo	KIF14	–	(61)
2020	HepG2, LM3, Huh7, SKhep1	Secondary	Sorafenib	*In vitro*, vivo	CircRNA-SORE	–	(62)
2020	HepG2	Secondary	Sorafenib	*In vitro*, vivo	circFN1	–	(63)
2020	PLC/PRF/5	Secondary	Cisplatin and doxorubicin	*In vitro*, vivo	NRF2, SHH	–	(64)
2021	HCCLM3	Secondary	Sorafenib	*In vitro*	PI3K/AKT	ITE	(65)
2021	Huh-7, Hep3B	Secondary	Doxorubicin	*In vitro*	LncRNA MALAT1	–	(66)
2021	Huh7, PLC/PRF/5	Secondary	Sorafenib	*In vitro*, vivo	NF-κB	CYP1A2	(67)
2021	HepG2, Huh7	Secondary	Lenvatinib	*In vitro*, vivo	VEGFR2	Sophoridine	(68)
2021	HA22T	Secondary	HDACi	*In vitro*	Cofilin-1	Platycodin D	(69)
2021	Huh7, PLC	Secondary	Sorafenib	*In vitro*, vivo	CBX4	–	(70)
2021	HepG2	Secondary	Sorafenib	*In vitro*, vivo	Midkine	UsLNPs	(71)
2021	Bel7402	Secondary	5-FU	*In vitro*, vivo	CDK1, cyclin B	CHC	(72)
2021	HepG2, Huh7	Secondary	Sorafenib	*In vitro*	CircFOXM1	–	(73)
2021	HepG2, Huh7	Secondary	Sorafenib	*In vitro*	YAP	–	(74)
2021	HepG2	Secondary	Sorafenib	*In vitro*, vivo	NgBR	Artesunate	(75)
2021	HepG2	Secondary	Doxorubicin	*In vitro*	Mitochondrial fuel dependence on glutamine	–	(76)
2021	HCCLM3	Secondary	Sorafenib	*In vitro*, vivo	TAK1	–	(77)
2021	SMMC-7721, Huh7	Secondary	Lenvatinib	*In vitro*, vivo	MT1JP	–	(78)
2021	HCCLM3, SK-Hep-1, HepG2	Secondary	Sorafenib	*In vitro*, vivo	UBQLN1	–	(79)
2021	HepG2	Secondary	Sorafenib	*In vitro*	ZFAS1	–	(80)
2021	HuH6, HepG2	Secondary	Doxorubicin	*In vitro*, vivo	USP8	–	(81)
2021	Huh7	Secondary	Sorafenib	*In vitro*	BAFF, NFκB	–	(82)
2021	Huh-7, HCC-LM3, Li-7	Secondary	Sorafenib	*In vitro*	WDR4	–	(83)
2021	Huh7, Hep3B	Secondary	Sorafenib	*In vitro*, vivo	HIF1α	–	(84)
2021	HepG2	Secondary	Doxorubicin	*In vitro*, vivo	–	NO-DOX@PDA-TPGS-Gal	(85)
2021	HepG2	Secondary	Oxaliplatin	*In vitro*	LINC01134	–	(86)
2021	PLC/PRF/5, Huh7	Secondary	Sorafenib	*In vitro*, vivo	HDAC4, MEF2D	–	(87)
2021	HepG2215, Hep3B	Secondary	Sorafenib	*In vitro*, vivo	YAP, IGF-1R	–	(88)
2021	Huh7	Secondary	Sorafenib	*In vitro*	FcRn	–	(89)
2021	Huh7	Secondary	Regorafenib	*In vitro*	Wnt and TGF-β Signalling	–	(90)
2021	HepG2	Secondary	Doxorubicin	*In vitro*, vivo	TGF-β, Smad	AANG	(91)
2021	MHCC-LM3, MHCC-97H, Hep3B, HepG2, Huh7	Secondary	Sorafenib	*In vitro*, vivo	RCN1	–	(92)
2021	Huh7, Hep3B, HLE	Secondary	Sorafenib	*In vitro*, vivo	YAP, TAZ, ATF4	–	(93)
2021	Huh7	Secondary	Sorafenib	*In vitro*	STAT3	–	(94)
2021	HepG2	Secondary	Doxorubicin, sorafenib, lenvatinib	*In vitro*, vivo	–	PS-ZL-7c aptamer	(95)
2022	MHCC97H, MHCC97L	Secondary	Ionizing radiation	*In vitro*, vivo	Integration of glucose and cardiolipin anabolism	–	(96)
2022	Huh7	Secondary	Sorafenib	*In vitro*	ZFAS1	–	(97)
2022	MHCC97L	Secondary	Sorafenib	*In vivo*	ETS1/miR-23a-3p/ACSL4 signalling	–	(98)
2022	Huh7	Secondary	Sorafenib	*In vitro*, vivo	–	DBPR114	(99)
2022	SNU-449, Hep3B	Secondary	Sorafenib	*In vitro*	USP22, ABCC1	–	(100)
2022	Huh-7, HepG2	Secondary	Sorafenib	*In vitro*	miR-10b-3p	–	(101)
2022	PLC/PRF/5, MHCC-97H	Secondary	Sorafenib	*In vitro*, vivo	SCAP	–	(102)
2022	Hep3B	Secondary	Sorafenib	*In vitro*	Autophagy	Fingolimod	(103)
2022	Huh7, Hep3B, HepG2	Secondary	Sorafenib	*In vitro*	Ets1	–	(104)
2022	HepG2	Secondary	Sorafenib	*In vitro*, vivo	EGFR	Fucoidan	(105)
2022	HuH7, PLC/PRF/5, Hep1-6	Secondary	Lenvatinib	*In vitro*, vivo	EGFR	–	(106)
2022	HepG2	Secondary	Sorafenib	*In vitro*	–	Ruthenium	(107)
2022	HuH-7, MHCC-97H	Secondary	Sorafenib	*In vitro*	MCM2	–	(108)
2022	Huh7, HepG2	Secondary	Sorafenib	*In vitro*, vivo	HDLBP	–	(109)
2022	MHCC97H, Hep3B, Hepa 1–6	Secondary	Oxaliplatin	*In vitro*, vivo	PD-L1, PMN-MDSC	–	(110)
2022	Hepa1-6	Secondary	Anti-PD-L1	*In vivo*	CD38	–	(111)
2023	Huh7, PLC/PRF/5, Hep3B	Secondary	Lenvatinib	*In vitro*, vivo	METTL1	–	(112)
2023	7,404	Secondary	Cisplatin	*In vitro*	–	Gal-NP@TPt	(113)
2023	Huh7	Secondary	Oxaliplatin, 5-FU	*In vitro*, vivo	5-hmC	–	(114)
2023	Huh7	Secondary	Sorafenib	*In vitro*	XPO1	–	(115)
2023	Huh-7, PLC	Secondary	Lenvatinib	*In vitro*	Curcumin	–	(116)
2023	Huh7	Secondary	Lenvatinib	*In vitro*, vivo	METTL3	–	(117)
2023	HepG2	Secondary	Cisplatin	*In vitro*, vivo	–	AR-NADR	(118)
2023	Huh7, Hep3B	Secondary	Lenvatinib, Sorafenib	*In vitro*, vivo	CAF-derived SPP1	–	(119)
2023	HCCLM3, Huh7	Secondary	Sorafenib	*In vitro*, vivo	Mitophagy	MenSCs	(120)
2023	Huh7	Secondary	Sorafenib	*In vitro*, vivo	CSNK1A1	–	(121)
2023	MHCC97H, PLC/PRF/5	Secondary	Sorafenib	*In vitro*, vivo	PLEKHG5	–	(122)
2023	Huh7	Secondary	Sorafenib	*In vitro*	MEX3A	–	(123)
2023	MHCC97H, MHCC97L	Secondary	Ionizing radiation	*In vitro*, vivo	Hexokinase 2	–	(124)
2023	Huh7, SK-Hep-1, Hep3B, HepG2	Secondary	Sorafenib	*In vitro*, vivo	Glycolysis-lactate metabolism	β-HB	(125)
2023	HUH7, PLC/PRF/5	Secondary	Sorafenib	*In vitro*, vivo	HSPB1	MiR-654-5p	(126)
2023	HepG2	Secondary	Apatinib	*In vitro*	RB1	–	(127)
2023	PLC/PRF/5, Huh7	Secondary	Lenvatinib	*In vitro*, vivo	CDK6	–	(128)
2023	Hep3B, MHCC97H, Hepa1-6	Secondary	Oxaliplatin	*In vitro*, vivo	NLRP3/IL-1β	–	(129)
2023	SMMC7721	Secondary	Sorafenib	*In vitro*, vivo	–	TME-responsive nano-platform	(130)
2023	Huh-7	Secondary	Sorafenib	*In vitro*	Galectin-1	–	(131)
2023	Huh7, SMMC-7721	Secondary	Sorafenib	*In vitro*, vivo	SMYD3	–	(132)
2024	Huh7, SK-Hep1	Secondary	Sorafenib	*In vitro*, vivo	SIRT7	–	(133)
2024	Hepa1-6	Secondary	Anti-PD-1	*In vivo*	CRKL	–	(134)
2024	MHCC97L	Secondary	Sorafenib	*In vitro*, vivo	LINC01056	–	(135)
2024	HepG2	Secondary	Sorafenib	*In vitro*, vivo	STAT3	STAT3 ASOs	(136)
2024	HepG2	Secondary	Cisplatin	*In vitro*, vivo	–	Glycyrrhetinic Acid	(137)
2024	BCLC-3	Primary	Sorafenib	*In vitro*, vivo	Metallothionein-3	–	(138)

### 2.2 Drug-resistant mechanisms based on traditional models

Li et al. (3) established sorafenib-resistant HCC (HepG2 and Huh7) cell lines and mouse models. To help Huh7-SR cells maintain their sorafenib-resistant ability, mice injected with Huh7-SR cells subcutaneously underwent daily treatment of sorafenib at a dose of 10 mg/kg. SR-HCC cells showed higher levels of lncRNA SNHG1 expression, miR-21 expression and Akt pathway activation than parental cells. SNHG1 activates the Akt pathway by regulating SLC3A2. Akt pathway inactivation induced by SNHG1 inhibition significantly increased the sensitivity of SR-HCC cells to sorafenib. Additionally, sorafenib induced the transfer of miR-21 to the nucleus, and miR-21 could continue to induce SNHG1 expression. Using the *in-vitro* and *in-vivo* sorafenib-resistant models, they found that LncRNA SNHG1 caused sorafenib resistance through activation of the Akt pathway. Reiter et al. (8) created a sorafenib-resistant HCC cell line to investigate the effect of ribociclib on the treatment of sorafenib-resistant HCC. HepG2 cells that were continuously incubated with sorafenib at escalating concentrations for 8 months, up to a final sorafenib concentration of 4 μM, were used as sorafenib-resistant cells and maintained in the medium with 4 μM sorafenib. They discovered that ribociclib reduced Rb expression and induced G1 cell cycle arrest in SR-HepG2 cells with Rb-high/p16-low protein expression profiles, indicating that ribociclib may be a good choice for the treatment of certain sorafenib-resistant HCC. Qiu et al. (13) established sorafenib-resistant HuH7 cells and xenograft mouse models and examined a group of patients with advanced, recurrent HCC to evaluate the clinical significance of sorafenib therapy. They found that high 14-3-3η expression and low miR-16 expression were related to sorafenib resistance and poor prognosis of HCC. Moreover, Chen et al. (36) initiated secondary sorafenib-resistant HuH-7 cells and xenograft mouse models. EphA2 was recognised as a crucial molecule in sorafenib resistance by quantitative phosphoproteomic analysis. It has been confirmed in *in vivo* animal models that sorafenib resistance can be successfully treated by concurrently inhibiting EphA2. Yang et al. (63) established sorafenib-resistant HepG2 cells and analysed the expression differences of circRNAs between sorafenib-sensitive and sorafenib-resistant HepG2 cells. CircFN1 was upregulated in sorafenib-resistant HepG2 cells and induced sorafenib resistance through the miR-1205/E2F1 signalling pathway. Zhao et al. (70) created sorafenib-resistant *in-vitro* and *in-vivo* models to explore the role of CBX4 in sorafenib resistance of HCC. CBX4 was upregulated in sorafenib-resistant Huh7 and PLC cells. Tumour growth could be suppressed by CA3- and UNC3866-mediated YAP1 and CBX4 inhibition *in vivo*. Younis et al. (71) designed an ultra-small lipid nanoparticle encapsulating sorafenib and midkine-siRNA and examined the effect of the new nanoparticles on treating sorafenib-resistant HCC with sorafenib-resistant cell-derived (HepG2) xenograft mouse models. The tumours in the xenograft models were eradicated by 70% using the nanoparticles, demonstrating the potential of this new approach in HCC treatment with sorafenib resistance. Xu et al. (79) constructed sorafenib-resistant *in-vitro* and *in-vivo* models to explore the role of UBQLN1 in HCC sorafenib resistance. For the sorafenib-resistant HCC mouse model, 100 million HCCLM3 cells were initially implanted into the flank of a BALB/c mouse. The formed tumours were re-implanted into 4-week-old BALB/c nude mice and fed with sorafenib (30 mg/kg/day). The mice that survived after 8 weeks of treatment were regarded as sorafenib-resistant mice. The ROS levels decreased in sorafenib-resistant HCC cells. However, sorafenib-resistant cells have better mitochondrial function and integrity with less mitochondrial content and respiratory capacity. Mechanically, these phenomena may be achieved by UBQLN1 through PGC1β inhibition in HCC. Fang et al. (96) established radiation-resistant HCC cell lines and xenograft mouse models to investigate the resistant mechanism of HCC to radiotherapy. MHCC97L cells were exposed to 8 Gy IR every 2 days for 5 fractions, and MHCC97H cells were exposed to 2 Gy IR daily for 25 fractions. After 4 weeks of recovery time, cells were again exposed to 10 Gy IR. MHCC97L IR-R cells were xenografted into nude mice and exposed to IR (8 Gy × 2 F) to establish radiation-resistant *in-vivo* models. Subsequent functional experiments demonstrated that the integration of glucose and cardiolipin anabolism was crucial for the radiation resistance of HCC. Zhou et al. (97) performed single-cell RNA sequencing in parental and sorafenib-resistant Huh7 cells using the 10X Genomic Chromium System. Huh7-R cells presented upregulations of stemness markers, EMT-related genes and Notch signalling-related genes, indicating that Notch signalling activation may be crucial for the induction of tumour stemness/EMT traits and acquired sorafenib resistance. Moreover, ZNFX1 antisense RNA 1 (ZFAS1), a new regulator IncRNA, had the highest upregulation in Huh7-R cells. Mechanically, the knockdown of ZFAS1 caused the downregulation of various mRNAs related to stemness and notch signalling pathways, indicating the critical role of this noncoding RNA in HCC sorafenib resistance. Huang et al. (112) created lenvatinib-resistant HCC cell lines and cell-derived xenograft mouse models. The two essential parts of the tRNA N7-methylguanosine (m7G) methyltransferase complex—methyltransferase-like protein-1 (METTL1) and WD repeat domain 4 protein (WDR4)—were significantly increased in lenvatinib-resistant cells. The crucial role that METTL1/WDR4-mediated m7G tRNA modification plays in developing lenvatinib resistance *in vivo* was further elucidated by xenograft mice models. Mechanically, METTL1 genes triggered drug resistance by EGFR pathway activation in HCC. Yang et al. (113) established the Pt-resistant HCC cell line 7404DDP to evaluate the antitumour effect of a cascade targeted and mitochondrion-dysfunctional nanomedicine (Gal-NP@TPt). When compared to cisplatin, Gal-NP@TPt caused a 9-fold increase in Pt accumulation in 7404DDP cells. Moreover, Gal-NP@TPt caused significant DNA damage in 7404DDP cells. Furthermore, Gal-NP@TPt may mitigate platinum resistance as the ratio of IC50 for 7404DDP to that for 7,404 dropped from 6.34 for cisplatin to 0.71 for Gal-NP@TPt. Kim et al. (133) established secondary sorafenib-resistant HCC cells and cell-derived mouse models to investigate the role of SIRT7 in sorafenib resistance. In Huh7SR and SK-Hep1SR cells, hyperactivated pERK1/2 was seen in conjunction with increased SIRT7 expression. *In-vivo* tumour development was suppressed by inhibiting SIRT7. Mechanically, SIRT7 inhibition eliminates sorafenib resistance by decreasing ERK1/2 phosphorylation via the DDX3X-mediated NLRP3 inflammasome in HCC.

## 3 Patient-derived drug resistance models

### 3.1 Establishment methods of patient-derived drug resistance models

**Figure 2:**
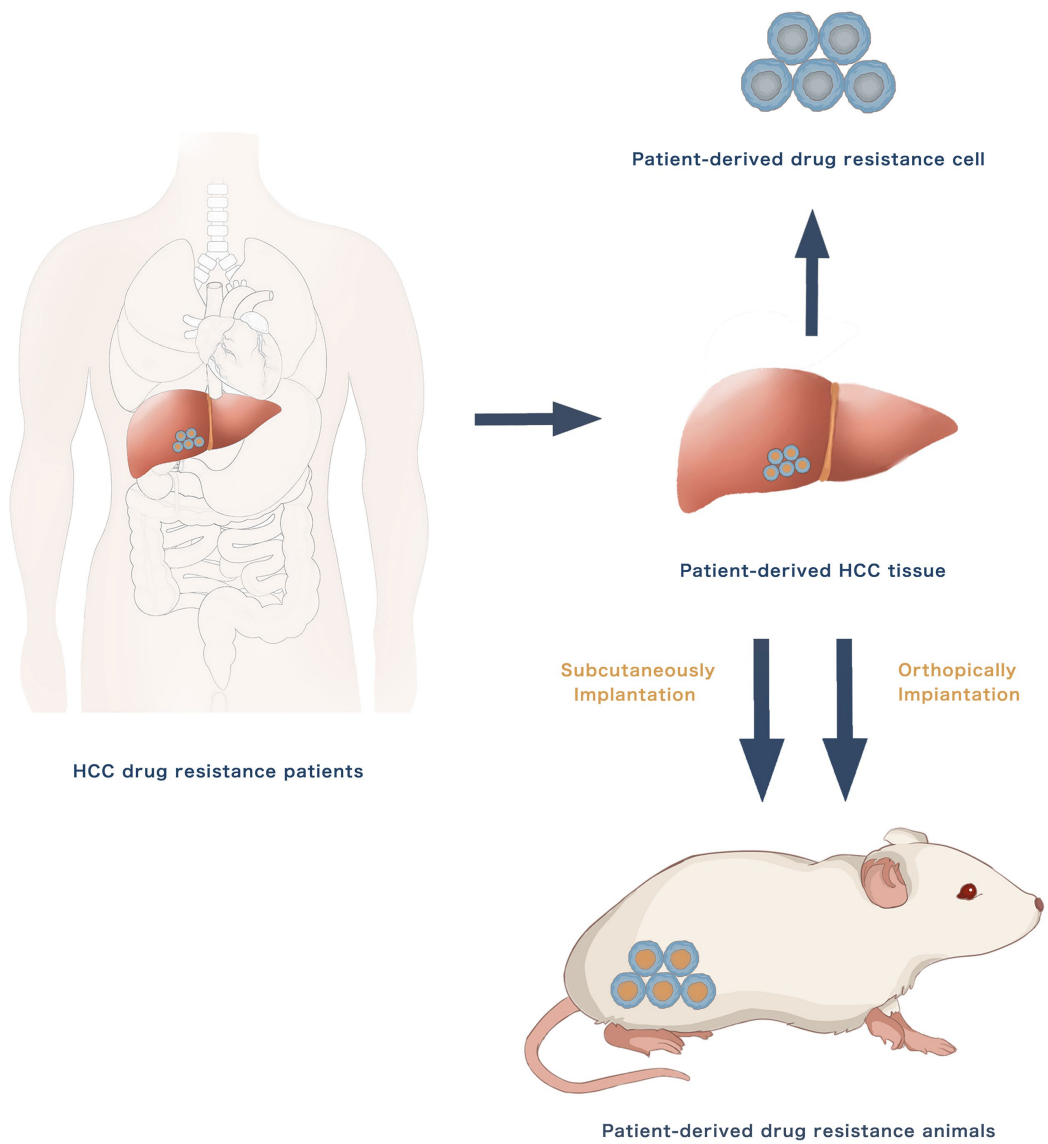
Hepatocellular carcinoma patient derived drug resistance models.

The use of patient-derived HCC cell lines and xenograft mouse models has increased in the study of HCC drug resistance. New drug-resistant cell lines may be created using primary cultures of HCC tissues from patients who have developed drug resistance. The resected tumour tissue samples may endure tissue digestion, cell separation and purification and primary cell culture to form a stable cell line. Patient-derived xenografts (PDXs) from patients with drug resistance could be directly implanted in nude mice subcutaneously or orthotopically to establish *in-vivo* drug resistance models. Figure 2 presents the establishment methods of patient-derived drug resistance models. The patient-derived drug resistance models better reserve individual molecular signature, including DNA copy number alterations, mutations and gene expression levels. The patient-derived drug resistance models may have additional advantages over the traditional models in the investigation of drug resistance mechanisms and individualised treatment as they can more accurately imitate the pathophysiological features of individual patients.

### 3.2 Drug-resistant mechanisms based on patient-derived models

Hu et al. (139) established sorafenib-resistant and sorafenib-sensitive PDX mouse models and examined the expression pattern differences between them. KPNA3 was found overexpressed in the sorafenib-resistant PDX models and was further confirmed to induce EMT and sorafenib resistance of HCC cells through KPNA3-AKT-ERK-TWIST signalling. Wang et al. (140) used HCC patient-derived organoid models to investigate the functions of Hedgehog signalling and CD44 in sorafenib resistance. In CD44-positive HCC, GANT61 dramatically reduced Hedgehog signalling to reverse sorafenib resistance, demonstrating that sorafenib with Hedgehog signalling inhibitors may be a useful therapeutic strategy for HCC patients with elevated CD44 levels. Hashiba et al. (33) revealed a patient-derived sorafenib-resistant HCC cell line (HCC-SR). The single-cell suspensions obtained from post-sorafenib HCC tissues were subcutaneously injected in nonobese diabetic, severe combined immunodeficient (NOD/SCID) mice, and the subcutaneous tumours were harvested to establish the new cell line. Sorafenib treatment induced higher cell proliferation of HCC-SR than other HCC cell lines, showing the resistance of HCC-SR to sorafenib. CIC S1595P missense mutation was detected in HCC-SR by whole-exome sequence analysis, revealing the potential function of this gene on HCC sorafenib resistance. Prawira et al. (141) established infigratinib-resistant PDX mouse models to explore the function of ribociclib in overcoming infigratinib resistance in HCC. SCID mice were subcutaneously implanted with four HCC PDX tumours with high FGFR1-4 expression, administered infigratinib (15 mg/kg per day) and sacrificed when tumours were 1800 mm^3^. Infigratinib-resistant tumours were re-implanted into SCID mice, and nine treatment cycles were required to maintain infigratinib resistance. Then, the infigratinib-resistant PDX mouse models were treated with either infigratinib alone or in combination with ribociclib and sacrificed for subsequent examination. The results showed that the combined inhibition of FGFR/CDK4/6 pathways is efficient in overcoming infigratinib resistance. Xu et al. (62) established four sorafenib-resistant HCC cell lines (HepG2-SR, LM3-SR, Huh7-SR, and SKhep1-SR) and cell-derived and patient-derived sorafenib-resistant xenograft mouse models to explore the mechanisms of sorafenib resistance in HCC. They found that circRNA-SORE was overexpressed in sorafenib-resistant HCC cells, induced sorafenib resistance and activated the Wnt/β-catenin pathway by sponging miR-660-3p and miR-103a-2-5p. Moreover, The cytoplasmic binding of circRNA-SORE to the master oncogenic protein YBX1 inhibits PRP19-mediated YBX1 degradation by blocking YBX1’s nuclear interaction with the E3 ubiquitin ligase PRP19 (142). Moreover, sorafenib resistance could be spread via exosomal circRNA-SORE transport (142). A local injection of circRNA-SORE shRNA lentivirus could significantly enhance the sensitivity of sorafenib treatment in mouse models. Liao et al. (143) investigated the potential of 17-AAG in overcoming sorafenib resistance using secondary sorafenib-resistant PDX mouse models. The HCC tissues from patients were transplanted into the armpit of severe NSG immunodeficient mice. Sorafenib (80 mg/kg) was administered orally to the mice once a day when the tumour size reached 100 mm^3^. The tumours were significantly resistant to sorafenib in the fourth generation. Additionally, 17-AAG suppressed HSP90α and reversed sorafenib resistance *in vivo*, indicating the potential of 17-AAG in overcoming sorafenib resistance. Leung et al. (144) created two sorafenib-resistant PDX mouse models, which resembled the emergence of sorafenib-induced acquired resistance in patients with HCC. The tumours from two HCC patients were xenografted into the immunodeficient mice, which were then administered with several cycles of sorafenib treatment to acquire sorafenib resistance. Two sorafenib-resistant HCC cell lines were further developed from the above two PDX models, with stronger self-renewal and tumourigenicity. RNA-sequencing recognised EPH receptor B2 (EPHB2) as the most significantly upregulated kinase in sorafenib-resistant PDXs. Functional experiments demonstrated that EPHB2 may increase tumour stemness and induce sorafenib resistance through the EPHB2/β-catenin/TCF1 positive feedback loop. Prawira et al. (145) established seven acquired infigratinib-resistant PDX models and developed a new infigratinib-resistant HCC cell line from one of these PDXs. Infigratinib-resistant tumours presented higher p-ErbB2, p-ErbB3 and EZH2 levels. Mechanically, EZH2 may promote infigratinib resistance by upregulating the ErbB family. Gao et al. (84) found high USP29, HIF1α and GLUT1 levels in sorafenib-resistant PDX tumours, and follow-up research revealed that USP29-induced sorafenib resistance by mediating HIF1α stabilisation and upregulated glycolysis. Mok et al. (146) used two drug-resistant tumour xenografts derived from HCC patients to investigate driving resistance and CSC repopulation in HCC. The xenografts mimicked the development of acquired resistance to sorafenib or lenvatinib treatment observed in HCC patients. RNA sequencing showed that cholesterol production was most frequently elevated in the drug-resistant xenografts. Mechanically, the drug resistance in HCC is driven by caspase-3-induced SREBP2 activation, which promotes cholesterol biosynthesis. Tao et al. (147) constructed six PDX models from HCC patients. The organoids from different patients showed different IC50 values of sorafenib treatment, indicating their sensitivity differences to sorafenib. BBOX1-AS1 was significantly upregulated in the organoids with the highest IC50 value, indicating that BBOX1-AS1 may be associated with sorafenib resistance. Ruan et al. (148) established a sorafenib-resistant PDX mouse model to investigate the function of a circular RNA, cDCBLD2. Tumour tissues from sorafenib-resistant HCC patients were implanted into the livers of NOD/SCID mice. Four weeks later, the mice were administered sorafenib at 30 mg/kg/d by gavage for 8 weeks to sustain sorafenib resistance. Then, the tissues were cut into equal pieces and re-implanted into the armpits of 4-week-old BALB/c nude mice, which were reared for subsequent *in-vivo* experiments. When *in vivo* grade cholesterol-conjugated si-cDCBLD2 was locally injected around the PDX implantation site, the sensitivity to sorafenib treatment was much higher than when a control siRNA was injected. *In-vitro* experiments further discovered that cDCBLD2-mediated sorafenib resistance was achieved by sponging miR-345-5p binding to the TOP2A coding sequence. Zhang et al. (149) found that YTHDF1 enhanced CSC renewal and resistance to the multiple tyrosine kinase inhibitors lenvatinib and sorafenib by upregulating NOTCH1 in patient-derived organoids and HCC cell lines. Additionally, Leung et al. (150) established sorafenib-resistant HCC cell lines and sorafenib-resistant PDX mouse models to evaluate the combined therapeutic effect of sorafenib and Src homology 2 domain-containing phosphatase 2 (SHP2) inhibitor on sorafenib-resistant HCC. NOD/SCID mice bearing PDX were orally administered sorafenib at 100 mg/kg/day for 25 days to acquire sorafenib resistance. SHP2 was significantly upregulated in sorafenib-resistant HCC cell lines and PDXs. Sorafenib combined with SHP2 inhibitor SHP099 showed high treatment efficacy in sorafenib-resistant PDX mice.

## 4 Direct detection of clinical drug-resistant samples from HCC patients

Peripheral blood and tumour tissues from HCC patients may be directly detected for gene expression levels using quantitative or semi-quantitative techniques such as qPCR, western blot or immunohistochemistry. In drug-resistant HCC samples, highly expressed genes are generally more likely to perform as drug resistance genes, whereas suppressed genes may be able to withstand drug resistance. Table 2 presents research on the direct detection of clinical drug-resistant samples from HCC patients.

**Table 2:** Researches on the direct detection of clinical drug-resistant samples from HCC patients.

Year	Model	Primary/ secondary	Resisted drug	Core molecule	Combined drug	Reference
2019	HCC tissues of patients	Primary	Oxaliplatin	CCN2/ MAPK/ Id-1	–	(7)
2019	HCC tissues of patients	Primary	Sorafenib	miR-16, 14-3-3η	–	(13)
2019	Circulating tumour DNA of HCC patients	Secondary	Fisogatinib	On-target FGFR4 kinase domain mutations	Gatekeeper-agnostic, pan-FGFR inhibitor	(19)
2020	Serum of HCC patients	Secondary	Sorafenib	MiR-30e-3p	–	(151)
2020	HCC tissues of patients	Secondary	Sorafenib	Capicua	–	(33)
2020	HCC tissues of patients	Primary	Sorafenib	HANR	–	(37)
2020	HCC tissues of patients	Primary	Sorafenib	SNGH16	–	(43)
2020	HCC tissues of patients	Primary	Adriamycin	CircFoxo3	–	(47)
2020	Clinical data of HCC patients	Primary	Sorafenib	–	Apatinib	(152)
2020	HCC tissues of patients	Primary	Sorafenib	KCNQ1OT1	–	(57)
2020	HCC tissues and blood of patients	Primary	TKIs	Mutations in the PI3K/MTOR pathway	–	(153)
2020	HCC tissues and blood of patients	Primary	Sorafenib	CircRNA-SORE	–	(62)
2021	HCC tissues and plasma of patients	–	TACE	MiR-125b	–	(154)
2021	HCC tissues of patients	Primary	Doxorubicin	LncRNA MALAT1	–	(66)
2021	HCC tissues of patients	Secondary	Sorafenib	NF-κB	CYP1A2	(67)
2021	HCC tissues of patients	Primary	Oxaliplatin	UCA1	–	(155)
2021	HCC tissues of patients	Primary	Sorafenib	circFOXM1	–	(73)
2021	HCC tissues of patients	–	Doxorubicin, sorafenib	Shc3	–	(156)
2021	HCC tissues of patients	Primary	Cisplatin	LINC00173	–	(157)
2021	HCC tissues of patients	Primary	Sorafenib	DDR2	–	(158)
2021	cDNA samples of HCC patients	–	sorafenib	MTBP	–	(159)
2021	HCC tissues of patients	Primary	Sorafenib	HDAC4, MEF2D	–	(87)
2021	HCC tissues of patients	Secondary	Atezolizumab, bevacizumab	Immune exclusion, tumour dedifferentiation	–	(160)
2021	HCC tissues of patients	Primary	Sorafenib	RCN1	–	(92)
2021	HCC tissues of patients	Primary	Sorafenib	YAP, TAZ, ATF4	–	(93)
2021	HCC tissues of patients	Primary	Anti-PD1 antibody	CircTMEM181	–	(161)
2021	HCC tissues of patients	Primary	Sorafenib	STAT3	–	(94)
2021	HCC tissues of patients	Primary	Oxaliplatin	lncRNA DUBR	–	(162)
2022	HCC tissues of patients	Primary	Ionizing radiation	Integration of glucose and cardiolipin anabolism	–	(96)
2022	HCC tissues of patients	Primary	Sorafenib	USP22, ABCC1	–	(100)
2022	HCC tissues of patients	Primary	Sorafenib	SCAP	–	(102)
2022	HCC tissues and blood of patients	Primary	Camrelizumab	MCT	–	(163)
2022	HCC tissues of patients	Primary	Sorafenib	HDLBP	–	(109)
2022	HCC tissues of patients	Primary	Sorafenib	CXCR2	–	(164)
2022	HCC tissues of patients	Primary	Oxaliplatin	PD-L1, PMN-MDSC	–	(110)
2022	HCC tissues of patients	–	Anti-PD1 antibody	PKCα/ZFP64/CSF1 axis	–	(165)
2023	HCC tissues and blood of patients	Primary	PD-1 ICB	Toll-like receptors-4	Anti-Δ42PD-1 antibody	(166)
2023	Serum of HCC patients	Primary	Atezolizumab Plus Bevacizumab	VEGF-D, ANG-2	–	(167)
2023	Clinical data of HCC patients	–	Sorafenib	–	Regorafenib Plus PD-1 Inhibitor	(168)
2023	HCC tissues of patients	Secondary	Sorafenib	Autophagy and biotransformation	–	(169)
2023	HCC tissues and blood of patients	Primary	Anti-PD1 antibody	CD10 + ALPL+ neutrophils	–	(170)
2023	HCC tissues of patients	Secondary	Lenvatinib plus anti-PD1 antibodies	MAIT cells	–	(171)
2023	HCC tissues of patients	Primary	Cabozantinib and nivolumab	HCC-CAF	–	(172)
2024	HCC tissues of patients	Primary	Sorafenib	DUSP4	–	(173)
2024	HCC tissues and blood of patients	–	Anti-PD1 antibody	Serum amyloid A	–	(174)
2024	HCC tissues and blood of patients	Primary	Anti-PD1 antibody	S100A9 + CD14+ monocytes	–	(175)

Circulating tumour DNA (ctDNA) sequencing is a minimally invasive method that enables the collection of repeat samples. Hatlen et al. (19) sequenced the ctDNA of acquired fisogatinib-resistant HCC patients in a fisogatinib phase I trial. Two patients with disease progression had mutations in the gatekeeper and hinge-1 residues in the FGFR4 kinase domain. Further, subsequent experiments using *in-vivo* and *in-vitro* secondary fisogatinib-resistant models demonstrated that acquired fisogatinib resistance was related to FGFR4 kinase domain mutations. Yu et al. (67) examined the HCC tissues of patients who experienced recurrence after primary HCC resection and sequential sorafenib treatment and found an inverse expression between CYP1A2 and NF-κB p65 in the sorafenib-naïve primary HCC compared with its paired sorafenib-experienced recurrence. Moreover, Weng et al. (73) divided patients who received two cycles of sorafenib treatment into the sorafenib-sensitive and sorafenib-resistant groups. RNA sequencing showed higher circFOXM1 levels in sorafenib-resistant HCC tissues. Functional experiments revealed circFOXM1-induced sorafenib resistance by upregulating MECP2 expression via sponging miR-1324. Ma et al. (87) discovered that transcriptional factor myocyte enhancer factor 2D (MEF2D) was overexpressed in sorafenib-resistant HCC cell lines and HCC specimens, indicating a poor prognosis for sorafenib-treated HCC patients. Mechanically, coupling HDAC4 with MEF2D may activate ERK by inhibiting SPRY4, causing sorafenib resistance of HCC. Wang et al. (160) examined the compared tumour tissues (pretreatment and post-progression samples) of one HCC patient with acquired resistance to a combined treatment of atezolizumab and bevacizumab who subsequently underwent surgical resection of the tumour. The number of CD8+ T cells in the tumour area and PD-L1 level in tumour-infiltrating immune cells were decreased in the drug-resistant HCC tissue. Additionally, the drug-resistant tissue presented more progenitor/hepatoblast features in the gene expression profile. The abovementioned results show that the acquired resistance to the combined treatment may be caused by the immune-excluded tumour microenvironment and tumour dedifferentiation. Lu et al. (161) found that circTMEM181 expression was upregulated in puncture biopsies of HCC tissues from anti-PD1 antibody-resistant patients compared to those from anti-PD1-sensitive patients. Furthermore, a high exosomal circTMEM181 level was associated with immunosuppression of microenvironment and anti-PD1 resistance in HCC. Mechanistically, exosomal circTMEM181 promoted CD39 expression by sponging miR-488-3p in macrophages. They further created macrophage-specific CD39 knockout mice and discovered that CD73 expression in HCC cells and CD39 expression in macrophages could impair the function of CD8 + T cells and induce anti-PD1 resistance by activating the eATP-adenosine pathway. Meng et al. (170) discovered that CD10 + ALPL+ neutrophils were more abundant in the tumour tissues of anti-PD-1-resistant patients than in those of anti-PD-1-sensitive patients. Mechanically, tumour cells secreted NAMPT, which reprogrammed CD10 + ALPL+neutrophils via NTRK1, keeping them immature and preventing their maturation and activation. This was how CD10 + ALPL+neutrophils were generated. Immunosuppressive CD10 + ALPL+ neutrophils further mediated ongoing T-cell exhaustion, which increased resistance to anti-PD-1 treatment in HCC.

## 5 Transgenic drug resistance models

### 5.1 Establishment methods of transgenic drug resistance models

Gene-editing techniques could be used in studies on mechanisms of drug resistance by direct insertion or knockout of drug-resistant genes in cells or animal models. An RNA-guided DNA endonuclease derived from the type II CRISPR bacterial immune system, namely clustered regularly interspaced short palindromic repeats (CRISPR)-associated protein 9 (Cas9), has been extensively employed as a highly effective gene-editing method because of its capacity to target novel genes through the simple modification of single-guide RNA (sgRNA) sequences (176). The precise Watson–Crick base pairing between Cas9’s guide RNA and the target DNA region and a direct contact between Cas9 and a short DNA protospacer adjacent motif are critical for the targeted-sequence specificity of Cas9 (177–180). The two Cas9 nuclease domains—HNH and RuvC—catalytically cleave the double-stranded DNA (179, 180). A shift in the reading frame caused by random insertions or deletions may result in mutations at the targeted locations (176). Homologous recombination with an introduced homologous donor DNA may be used to complete homology-directed repair (181, 182).

### 5.2 Drug-resistant mechanisms based on transgenic models

The transcription factor called hepatic leukaemia factor (HLF) is a member of the proline and acidic amino acid-rich family, which are known to regulate circadian rhythms (183). Xiang et al. (184) constructed an HLF-knockout mouse model (HLFPB/PB mouse) and found that sorafenib resistance requires HLF-mediated c-Jun activation, demonstrating that the HLF-c-Jun axis may control the haepatoma’s response to sorafenib. A piggyBac transposon-encoding CAG-RFP (a red fluorescent protein sequence under the CAG promoter) was inserted into the HLF gene to create the HLFPB/PB mice. Diethylnitrosamine was injected into the abdomen of mice to induce haepatoma development. The mice were then euthanised 7, 8 or 9 months later. Haepatoma cells that overexpressed HLF became resistant to growth inhibition and cell death caused by sorafenib. The c-Jun interference eliminated sorafenib resistance in haepatoma cells overexpressing HLF. Furthermore, HLF interference weakened c-Jun activation due to sorafenib and made haepatoma cells more susceptible to sorafenib. Wei et al. (185) found phosphoglycerate dehydrogenase (PHGDH) to be a critical sorafenib-resistance gene by genome-wide CRISPR/Cas9 library screening. The Human GeCKOv2A CRISPR knockout pooled library contains 65,386 sgRNAs targeting 19,052 human genes and 1864 miRNAs. They constructed a stable Cas9-expressing HCC cell line (MHCC97L-Cas9) by lentiviral transfection and transfected it with the GeCKOv2A library. Mutant cells were selected by puromycin and treated with sorafenib and DMSO for a week and were collected. The sgRNAs were amplified by PCR for subsequent high-throughput sequencing and bioinformatic analysis. PHGDH targeting sgRNAs were negatively selected after sorafenib treatment. They further established CRISPR/Cas9 knockout and RNAi knockdown cell models and found that inhibition of PHGDH promotes HCC apoptosis by suppressing the synthesis pathway upon sorafenib treatment. Sueangoen et al. (186) developed transgenic cell models to evaluate the functions of seven HCC-derived EGFR mutations on erlotinib resistance. Seven missense mutations (K757E, N808S, R831C, V897A, P937L, T940A and M947T) in the kinase domain of EGFR were recognised and retrovirally transducted into NIH-3 T3 cells. T790M and L858R were used as erlotinib-resistant and erlotinib-sensitive mutant controls, respectively. *In vitro* experiments showed that the seven EGFR mutants are dependent on EGF and resistant to erlotinib. Sofer et al. (187) performed a genome-wide CRISPR/Cas9 activation screen in Huh7 and recognised hexokinase 1 as a critical factor in promoting regorafenib resistance in HCC. Moreover, Chen et al. recognised FGF21 as a sorafenib-resistant gene in HCC by CRISPR/CAS9 genome library screening. Mechanically, FGF21 induces sorafenib resistance by directly combining with NRF2 to prevent NRF2 ubiquitination degradation. Huang et al. (188) used CRISPR/CAS9 genome library screening and discovered that lenvatinib resistance was mediated by DUSP4 deficiency through the activation of MAPK/ERK signalling. Another gene associated with lenvatinib resistance, LAPTM5, was identified by Pan et al. (189). MiR-3689a-3p was found to be the most upregulated miRNA in sorafenib-sensitive HCC by CRISPR/Cas9 screens (190). Mechanically, miR-3689a-3p may suppress CCS/SOD1-dependent mitochondrial oxidative stress to regulate sorafenib resistance. Zhu et al. (191) used a transgenic HCC mouse model driven by MYC overexpression and beta-catenin (encoded by CTNNB1) activation (termed MYC-lucOS; CTNNB1) (192), which shows resistance to anti-PD-1 immunotherapy, to evaluate the effect of the combination of anti-PD-L1 immunotherapy and anti-VEGF. After 4 weeks of the combined treatment, a significant survival improvement and a decline in the proportion of mice with tumours were observed in this model, indicating the potential of the anti-PD-L1/anti-VEGF dual treatment in overcoming resistance to either of a single agent. Martin et al. (193) used CRISPR/Cas9 to directionally knock out tumour suppressor genes in HCC cells. The p53-knockout HepG2 cells performed increased malignant properties and multidrug resistance to cisplatin, regorafenib, sorafenib and doxorubicin. Alb-R26Met mice carry a conditional mouse–human chimeric Met transgene into the Rosa26 locus. The Alb-R26Met HCC is resistant to sorafenib. ADAMTSL5 overexpression was noted at the early stages of liver carcinogenesis, and its upregulation was reproduced in the Alb-R26Met HCC model. Various oncogenic inputs associated with HCC decreased as a result of ADAMTSL5 abrogation, such as MET, EGFR, PDGFRβ, IGF1Rβ and FGFR4 receptor tyrosine kinases, which were all expressed and/or phosphorylated to a lesser extent, showing the potential role of ADAMTSL5 in HCC drug resistance (194).

## 6 Summary

To further understand drug resistance mechanisms in HCC, useful models must be developed. Different kinds of models have their own advantages and disadvantages. Traditional drug resistance models are easier to establish. Meanwhile, the corresponding experimental results have higher stability and reproducibility. However, individual differences in HCC gene expression could not be reflected in a single cell line. Patient-derived models maintain more individual traits, which are essential for studying the various drug resistance pathways related to distinct clinical subtypes of cancer. Despite the difficulty of the construction process, patient-derived models may be a better choice to investigate the drug resistance mechanisms of various malignancies. Sometimes a direct detection of clinical drug-resistant samples may be a simpler method to screen for drug-resistant genes. Gene-editing methodologies can be employed to generate genetically engineered cell lines or animal models that exhibit resistance to particular pharmaceutical agents. Theoretically, a gene-editing cell line may be an ideal model to investigate the function of a specific gene on drug resistance. It has to be admitted that none of the aforementioned techniques is flawless. In the actual research, we should choose the appropriate drug resistance model according to the research purpose and the realistic research environment.

## Author contributions

YX: Writing – original draft. JW: Writing – original draft. HQ: Writing – review & editing.
